# A novel MRI diffusion metric ‘slow diffusion coefficient’ (SDC) for diagnosing isocitrate dehydrogenase (IDH) genotype in diffuse gliomas: initial promising results

**DOI:** 10.1186/s12880-025-01980-y

**Published:** 2025-10-27

**Authors:** Wan-Yi Zheng, Yu-Ting Shi, Ying-Ying He, Ruo-Lan Lin, Ben-Heng Xiao, Ri-Feng Jiang, Yì Xiáng J. Wáng

**Affiliations:** 1 Department of Radiology, Fujian Medical University Union Hospital, NO. 29 Xinquan Road, Fuzhou, Fujian 350001 P. R. China; 2 School of Medical Imaging, Fujian Medical University, Fuzhou, 350004 China; 3 Faculty of Medicine, Department of Imaging and Interventional Radiology, The Chinese University of Hong Kong, Shatin, New Territories, Hong Kong SAR China

**Keywords:** Diffusion magnetic resonance imaging, Glioma, Isocitrate dehydrogenase, Ki-67

## Abstract

**Background:**

Determining isocitrate dehydrogenase (IDH) mutation is crucial for glioma management. Slow diffusion coefficient (SDC) is a novel metric being proposed to measure in vivo tissue slow diffusion. In its basic form, SDC is derived from a high *b*-value diffusion-weighted image and a higher *b*-value diffusion-weighted image. The study attempts to distinguish IDH genotypes of diffuse gliomas using SDC alone and in combination with other two diffusion metrics of diffusion-derived vessel density (DDVD) and apparent diffusion coefficient (ADC).

**Methods:**

This study enrolled 63 patients with diffuse gliomas (30 IDH-mutant and 33 IDH-wildtype) who underwent diffusion-weighted imaging at 3T. SDC was calculated with *b* = 500 and 750 mm^2^/s images. DDVD was calculated with *b* = 0 and 10 mm^2^/s images. ADC was calculated with *b* = 0 and 1000 mm^2^/s images. Correlations between the diffusion metrics and IDH genotypes were studied, as well as the correlation between SDC and Ki-67 expression.

**Results:**

There was a significant difference among three histological grading of glioma (median value: 0.472 au/s for grade-2, 0.441 au/s for grade-3, 0.364 au/s for grade-4, *p* < 0.0001). Based on IDH gene testing, IDH mutant negative tumors had SDC value of 0.339 ± 0.055 au/s, IDH mutant positive tumors had SDC value of 0.437 ± 0.097 au/s, with AUROC of 0.828 for separation. SDC was negatively and weakly correlated with DDVD, with Pearson *r* of -0.212 (*p* = 0.096). A combination of SDC and DDVD separated IDH mutant -/+ tumors with an AUROC of 0.886. SDC was positively and moderately correlated with ADC, with Pearson *r* of 0.705 (*p* < 0.0001). AUROC analysis shows a combination of SDC, DDVD, and ADC separated IDH mutant -/+ tumors with an AUC of 0.897. If immunohistochemistry IDH partially positive tumors were not included, a combination of SDC, DDVD and ADC separated immunohistochemistry IDH mutant -/+ tumors with an AUC of 0.911. SDC was negatively and moderately correlated with Ki 67 LI, with Pearson *r* of -0.382 (*p* = 0.002).

**Conclusion:**

SDC can help to distinguish IDH genotypes in diffuse gliomas. A combination of SDC and DDVD, or a combination of SDC, DDVD, and ADC, can further improve disease classification.

**Clinical trial number:**

Not applicable.

## Background

Based on the molecular characteristics, particularly isocitrate dehydrogenase (IDH) status, adult-type diffuse gliomas are categorized into glioblastoma (IDH-wildtype), astrocytoma (IDH-mutant), and oligodendroglioma (IDH-mutant and 1p/19q-codeleted) [1]. IDH mutations are associated with better prognosis and therapeutic responses compared to IDH-wildtype gliomas. Molecular characterization has become critical in guiding treatment strategies and predicting outcomes for glioma patients.

Diffusion weighted imaging (DWI) has been tested to assess tumor grades and IDH genotypes of gliomas, with initial promising results [2–6]. Recently, magnetic resonance diffusion-derived ‘vessel density’ (DDVD), a functional parameter indicative of microvascular perfusion [7–9], has been tested for IDH genotyping in diffuse gliomas [10]. It was shown that DDVD was lower among IDH-mutant positive gliomas than among IDH-wildtype gliomas, with an AUC of 0.823 for separating IDH-mutant positive gliomas and IDH-wildtype gliomas [10]. Though the diffusion metric apparent diffusion coefficient (ADC) has been considered to reflect tissue cellularity, recent work suggests ADC measure is also contributed by tissue T2 relaxation time [11, 12]. This is partly due to that the high *b*-value imaging has a longer effective TE than the low *b*-value imaging [13], and short T2 tissues (commonly considered to be less ‘watery’) have faster signal decay between low *b*-value imaging and high *b*-value imaging. This is particularly problematic for tissues with relatively shorter T2 (those of less than 70 ms), such as cartilage showing a high ADC value (around 1.5 × 10^− 3^ mm^2^/s) and muscles showing a higher ADC than the liver (1.55 × 10^− 3^ mm^2^/s vs. 1.07 × 10^− 3^ mm^2^/s) [14]. To mitigate this, a novel metric slow diffusion coefficient (SDC) has been proposed [15]. In its basic form, SDC is derived from a high *b*-value DWI image and a higher *b*-value DWI image. With the conventional approach, the spleen has been reported to have a much lower ADC than liver, hepatocellular carcinomas have a lower ADC than liver parenchyma, and simple liver cysts have a higher ADC than liver hemangiomas. On the other hand, with SDC analysis, Xu et al. [15]. reported that the spleen has a faster diffusion than liver, hepatocellular carcinomas (HCCs) have a faster diffusion than liver parenchyma, and liver hemangiomas have a faster diffusion than simple liver cysts. The liver and spleen have a similar amount of blood perfusion, the spleen is waterier than the liver, and the spleen tissue has a higher contrast-enhanced CT extracellular volume fraction than the liver [16, 17]. HCCs are mostly associated with increased blood supply and increased proportion of arterial blood supply and with edema. It is more reasonable with SDC results that spleen and HCC have a faster diffusion than liver parenchyma. Due to the ‘flushing’ of blood flow inside the hemangioma, it is also more reasonable with SDC results that the diffusion of hemangioma liquid is faster than the more ‘static’ liquid of the cysts. Moreover, abscess liquid has been reported to be of low ADC with diffusion restriction which appears to be unreasonable [18]. However, SDC measure suggests that abscess liquid may have fast diffusion [19].

In this study, we hypothesize that the novel DWI metric of SDC may help the MRI-based separation of IDH-mutant positive gliomas and IDH-wildtype gliomas (IDH-mutant negative gliomas). Moreover, we tested whether a combination of the three DWI metrics (SDC, DDVD, and ADC) can further improve the MRI-based separation of IDH-mutant positive and negative gliomas. The generation of these three parameters only requires the imaging of four *b*-values, which is substantially faster than standard IVIM imaging.

## Methods

### Patients and clinical information

**Fig. 1:**
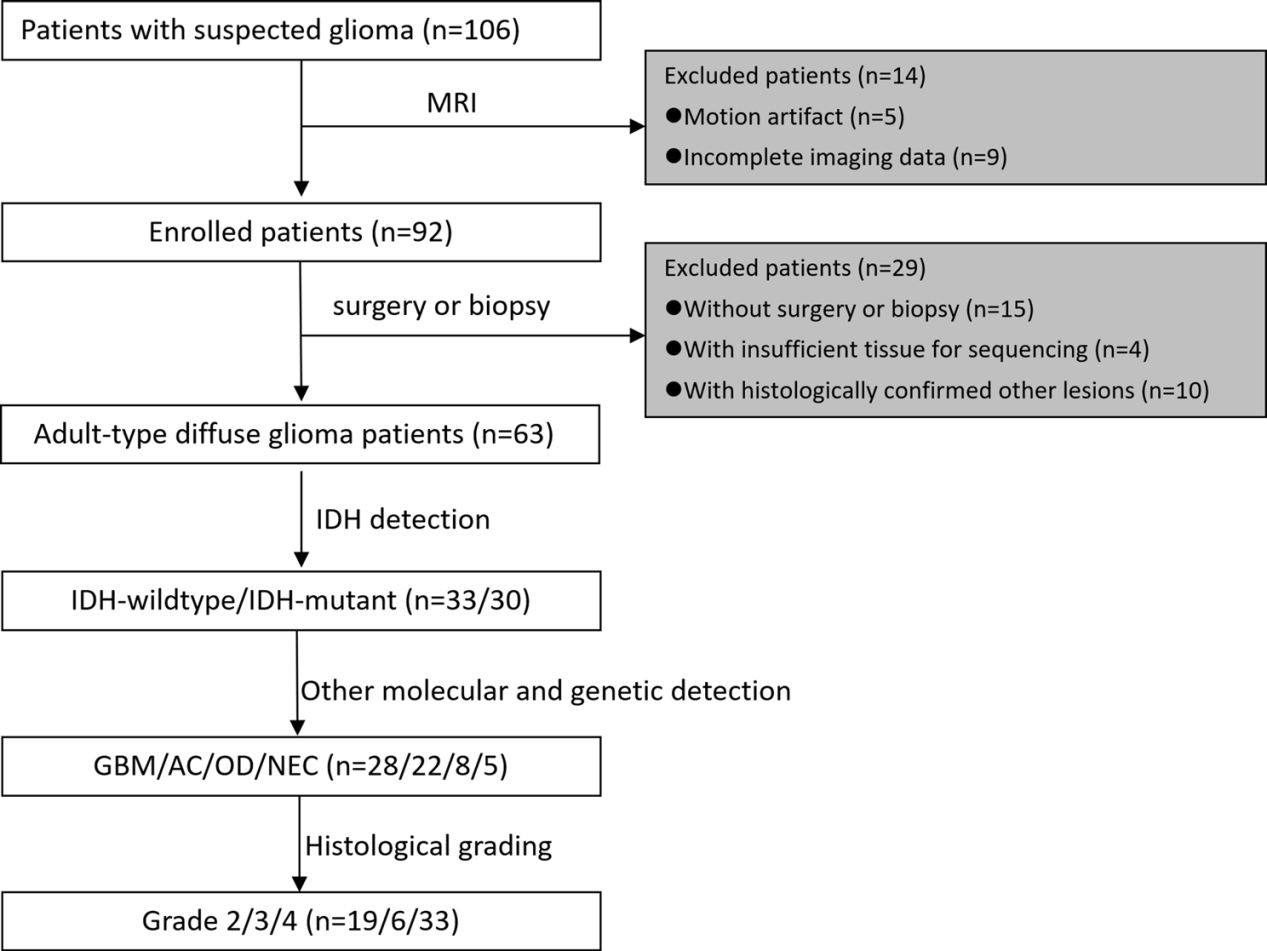
Glioma patient enrolment flow diagram. IDH = isocitrate dehydrogenase, GBM = glioblastoma; AC = astrocytoma; OD = oligodendroglioma; NEC = not elsewhere classified (these gliomas are not included in histological grading)

The patient data were the same as our recent article [10]. Ethical approval was obtained with the local Ethics Committee, and all participants signed informed consent. Between March 2019 and July 2023, this prospective study initially enrolled 106 glioma patients. Exclusion criteria included incomplete or poor-quality imaging, lack of pathohistological diagnosis, non-glioma histological diagnoses, inadequate tissue for genetic analysis, and prior glioma therapy. The final study cohort had 63 patients (33 males, 30 females) (Fig. 1).

### MRI data acquisition

MR imaging was conducted on a 3.0-T system (MAGNETOM Prisma, Siemens Healthcare) utilizing a 64-channel head coil [10]. The Prescan Normalize option was enabled to mitigate intensity inhomogeneity in MR images acquired with the 64-channel head coil, ensuring consistent signal intensity across both peripheral and central brain structures. Structural MRI included pre-contrast sagittal T1-weighted (T1W) images, axial T2-weighted (T2W) fast spin-echo (FSE) images, axial fluid-attenuated inversion recovery (FLAIR) T2W images, axial T2W gradient echo (GRE) sequence, and post-Gadolinium FLAIR T1W images in axial, sagittal, and coronal planes. For DWI, a single-shot echo planar imaging sequence was applied with the following parameters: repetition time, 2,000 ms; echo time, 69 ms; slice thickness, 5 mm; inter-slice gap, 1 mm; field of view, 23 cm × 23 cm; acquisition matrix, 128 × 128; GRAPPA, 2; slice acceleration factor, 2; and pixel bandwidth, 2055 Hz/pixel. The acquisition encompassed a series of 16 *b*-values (0, 10, 20, 40, 60, 80, 100, 120, 140, 160, 180, 200, 300, 500, 750 and 1,000 s/mm^2^) with a single number of excitation (NEX). DWI Images of *b* = 0 s/mm^2^ and four non-zero *b*-values (10, 500, 750 and 1,000 s/mm^2^) were utilized in the current study.

### DWI processing and analysis

SDC pixelwise maps were computed using the following equation [15]:

1 $$\begin{aligned}&{\text{SDC}}\,{\text{ = }}\,\left[ {S({b_1})-S({b_2})} \right]/({b_2}-{b_1}) \cr&[unit:\,arbitrary\,unit\,(au)/s]\end{aligned}$$

where *b*_*1*_ and *b*_*2*_ refer to a high *b*-value (500 mm^2^/s in this study) and a higher *b*-value respectively (750 mm^2^/s in this study), where S(*b*_*1*_) and S(*b*_*2*_) denote the image signal-intensity acquired at the high *b*-value and the higher *b*-value respectively.

DDVD was computed using the following equation [7, 9]:

2 $$\begin{gathered}{\text{DDV}}{{\text{D}}_{{\text{b0b10}}}}{\text{ = }}{{\text{S}}_{{\text{b0}}}}{\text{/RO}}{{\text{I}}_{{\text{area\_b0}}}} \hfill \\{\text{--}}{{\text{S}}_{{\text{b10}}}}{\text{/RO}}{{\text{I}}_{{\text{area\_b10}}}}{\text{}}\left( {{\text{au/pixel}}} \right) \hfill \\\end{gathered}$$

**Fig. 2:**
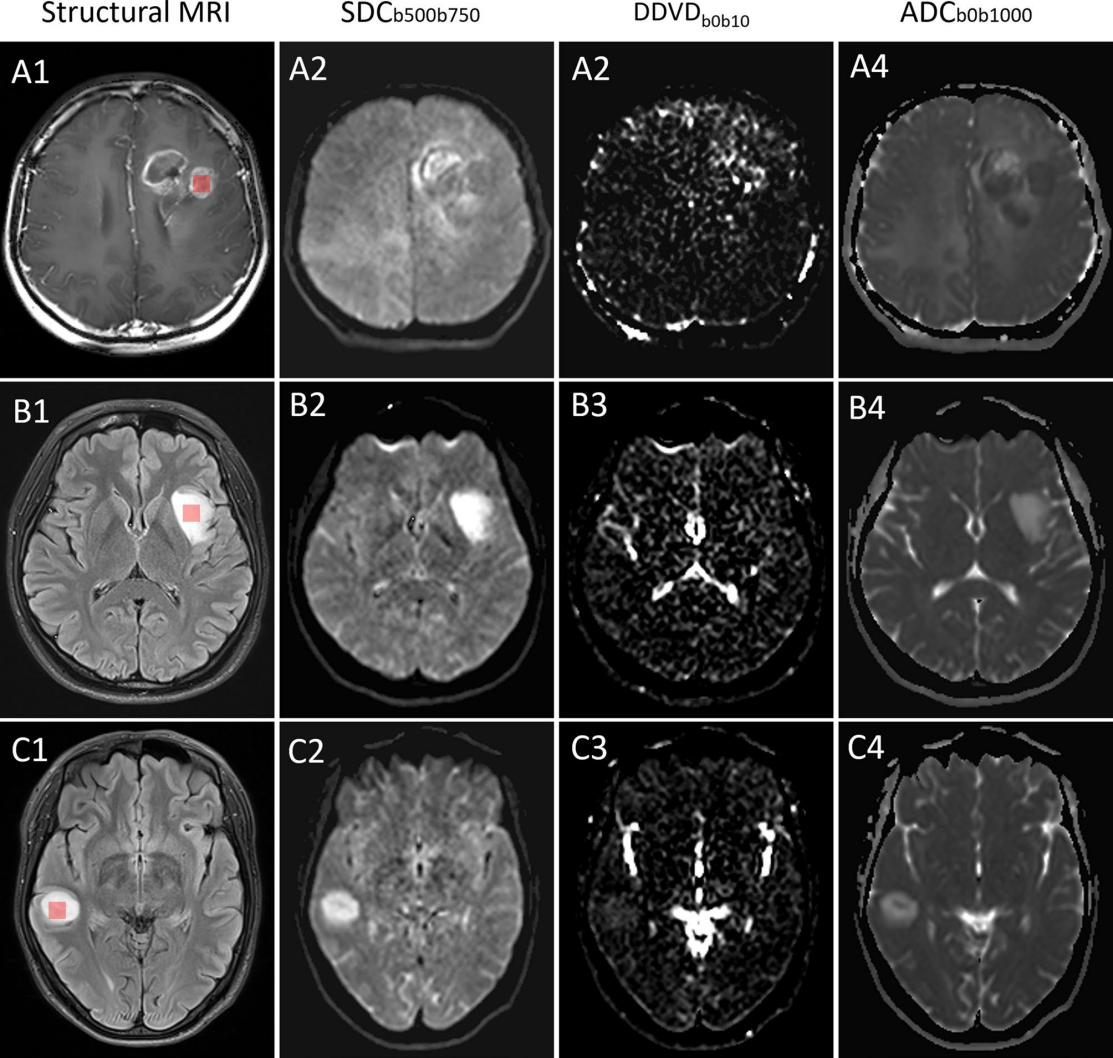
Examples of ROI delineation and representative cases of gliomas. (**A**) A patient with glioblastoma IDH-wildtype in the left frontal lobe (**A1**), showing relatively low SDC value (**A2**), high DDVD value (**A3**), and low ADC value (**A4**). (**B**) A patient with astrocytoma IDH-mutant (grade 2) in the left insular lobe (**B1**), showing high SDC value (**B2**), low DDVD value (**B3**), and high ADC value (**B4**). (**C**) A patient with astrocytoma IDH-mutant (grade 3) in the right temporal lobe (**C1**), showing high SDC value (**C2**), low DDVD value (**C3**), and relative high ADC value (**C4**). ROI = region of interest, IDH = isocitrate dehydrogenase, DDVD = diffusion-derived vessel density, SDC = slow diffusion coefficient

Where S_b0_ and S_b10_ refer to the sum of signals within the selected region of interest (ROI) on *b* = 0 and 10 s/mm^2^ images, respectively. ROI_area_b0_ and ROI_area_b10_ refer to the ROI area (unit in pixel) on *b* = 0 and 10 s/mm^2^ images. For DDVD pixelwise maps, DDVD_b0b10_ denotes the average signal intensity difference between *b* = 0 s/mm^2^ and *b* = 10 s/mm^2^, per pixel (Fig. 2).

Based on the ROI drawn for DDVD analysis, conventional ADC was calculated according to:

3 $$\begin{aligned}&ADC=\frac{{ln}(S\left({b}_{1}\right)/S\left({b}_{2}\right))}{{b}_{2}-{b}_{1}}\,\cr&[unit:{\text{ }}m{m^2}/s]\end{aligned}$$

where *b*_*2*_ and *b*_*1*_ refers to the high *b*-value (1000 mm^2^/s in this study) and low *b*-value (0 mm^2^/s in this study) respectively, where S(*b*_*2*_) and S(*b*_*1*_) denote the image signal-intensity acquired at the high *b*-value and low *b*-value respectively.

### ROI placement

All diffusion-weighted images were subjected to denoising and motion correction using QSIprep (https://github.com/PennLINC/qsiprep). MR images were registered to the *b* = 0 images using SPM12 (Wellcome Centre for Human Neuroimaging, UK). Blinded to histological results, ROIs of 310mm^3^ in size over the solid tumor parenchyma were delineated on a representative tumor slice using ITK-SNAP (version 3.8, NIH; https://www.itksnap.org) by two neuroradiologists (R.F.J. and Y.F.S.). The enhanced tumor parenchyma was outlined using transverse Gadolinium-enhanced T1-weighted images when clear enhancement region was present, while for non-enhanced tumors, the tumor parenchyma was identified on transverse T2-weighted fluid attenuated inversion recovery (FLAIR) images or T2-weighted fast spin echo images. Areas with identifiable necrosis, cysts, edema, and calcifications were excluded from the analysis. Figure 2 illustrates ROI delineation in sample cases for each tumor type.

### Inter-reader agreement

For a random selection of 20 glioma patients, the measurement was conducted twice. The intraclass correlation coefficient (ICC) was 0.922 (95% CI: 0.814–0.968) for SDC measurement, 0.832 (0.623–0.930) for DDVD measurement, and 0.921 (95%0.811–0.968) for ADC measurement. The measurement data from R.F.J were used for subsequent statistical analysis.

### Molecular and genetic detection of adult-type diffuse glioma

Molecular profiling was conducted via multiplexed polymerase chain reaction (PCR) coupled with next-generation sequencing to elucidate key genetic characteristics. Targeted detection of single-nucleotide variants (SNVs) identified mutations at IDH1 R132, IDH2 R172, TERT C228T, and TERT C250T. Copy number variation (CNV) analysis pinpointed 1p/19q co-deletion, chromosome 10 loss, and chromosome 7 gain. Quantitative real-time PCR evaluated EGFRvIII amplification. The presence of an IDH1 R132 or IDH2 R172 mutation classified the glioma as IDH-mutant; absence indicated an IDH-wildtype diagnosis.

Patients were grouped according to IDH genotypes (IDH-wildtype or IDH-mutant), and were subsequently divided into three tumor subtypes (glioblastoma, IDH-wildtype, astrocytoma, IDH-mutant and oligodendroglioma, IDH-mutant and 1p/19q-codeleted) and three grades (grade 2, 3 and 4) according to WHO CNS5 subtype criteria [1]. The Envision technique was used for immunohistochemical staining. The Ki-67 labeling index (LI) was defined as the percentage of nuclear staining-positive cells with any intensity in the high-density staining area in the total cells.

### Statistical analysis

Statistical analysis was conducted with GraphPad (San Diego, CA, USA). Group comparisons were conducted utilizing the Mann-Whitney U test or Kruskal-Wallis test for continuous variables. Receiver operating characteristic curve (ROC) and area under curve (AUC) were used to determine the diagnostic performance. Pearson correlation examined correlations among diffusion metrics as well as correlation between SDC and Ki-67 LI.

## Results

Patient demographics for the 63 subjects are detailed in Table 1. Of the 33 with IDH-wildtype glioma, 28 were glioblastoma and 5 were IDH-wildtype glioma, not elsewhere classified (NEC). The 30 with IDH-mutant glioma comprised 22 astrocytomas and 8 oligodendrogliomas. Histological grade-2 has 19 cases, grade-3 has 6 cases, and grade-4 has 33 cases with NEC glioma not graded.

**Table 1:** Participant demographic, clinical, and pathological characteristics

Characteristic	IDH-wildtype (*n* = 33)	IDH-mutant (*n* = 30)		*p*
		1p19q-intact (*n* = 22)	1p19q-codel (*n* = 8)	
Age (years)	57.5 (50–64)	36 (32–46)	45 (35–47)	< 0.001^**^
Sex (male/female)	17/16	12/10	4/4	0.469
KPS	80 (50–90)	80 (70–90)	90 (90–90)	0.038^*^
Recurrent/primary glioma	7/26	6/16	2/6	0.845
WHO CNS5 subtype				
Glioblastoma	28	NA	NA	NA
Astrocytoma (grade 2/3/4)	NA	13/4/5	NA	NA
Oligodendroglioma (grade 2/3)	NA	NA	6/2	NA
Ki-67 LI (%)	32.5 (12.5–50)	10 (5–30)	5 (4.5–10)	0.002^**^

Values are presented as number or median (interquartile range). ^*^ *p* < 0.05 and ^**^ *p* < 0.01. IDH = isocitrate dehydrogenase, 1p19q-codel = synchronous deletion of the short arm of chromosome 1 and long arm of chromosome 19, KPS = Karnofsky performance status, WHO CNS5 = fifth edition of the World Health Organization Classification of Tumors of the Central Nervous System, NA = not applicable, Ki-67 LI = Ki-67 labeling index

**Table 2:** ROC results of the performance of SDC in diagnosing IDH mutant status for glioma. For IDH mutant status diagnosed by immunohistochemistry, partial positive cases were removed

	SDC (au/s)	Specificity	95% CI	Sensitivity	95% CI	Likelihood ratio
IDH gene testing	> 0.3832	75.76%	58.98% to 87.17%	76.67%	59.07% to 88.21%	3.163
	> 0.3918	81.82%	65.61% to 91.39%	73.33%	55.55% to 85.82%	4.033
	> 0.3972	84.85%	69.08% to 93.35%	70%	52.12% to 83.34%	4.62
	> 0.4044	90.91%	76.43% to 96.86%	70%	52.12% to 83.34%	7.7
	> 0.4294	96.97%	84.68% to 99.84%	60%	42.32% to 75.41%	19.8
IDH immunohistochemistry	> 0.3832	74.29%	57.93% to 85.84%	86.67%	62.12% to 97.63%	3.370
	> 0.3940	80.00%	64.11% to 89.96%	80.0%	54.81% to 92.95%	4.000
	> 0.3998	85.71%	70.62% to 93.74%	80.0%	54.81% to 92.95%	5.600
	> 0.4202	91.43%	77.62% to 97.04%	73.33%	48.05% to 89.10%	8.556
	> 0.4439	97.14%	85.47% to 99.85%	73.33%	48.05% to 89.10%	25.67

SDC was negatively and moderately correlated with Ki 67 LI, with Pearson *r* of -0.382 (95% CI: -0.576 ~-0.149, *p* = 0.002).

**Fig. 3:**
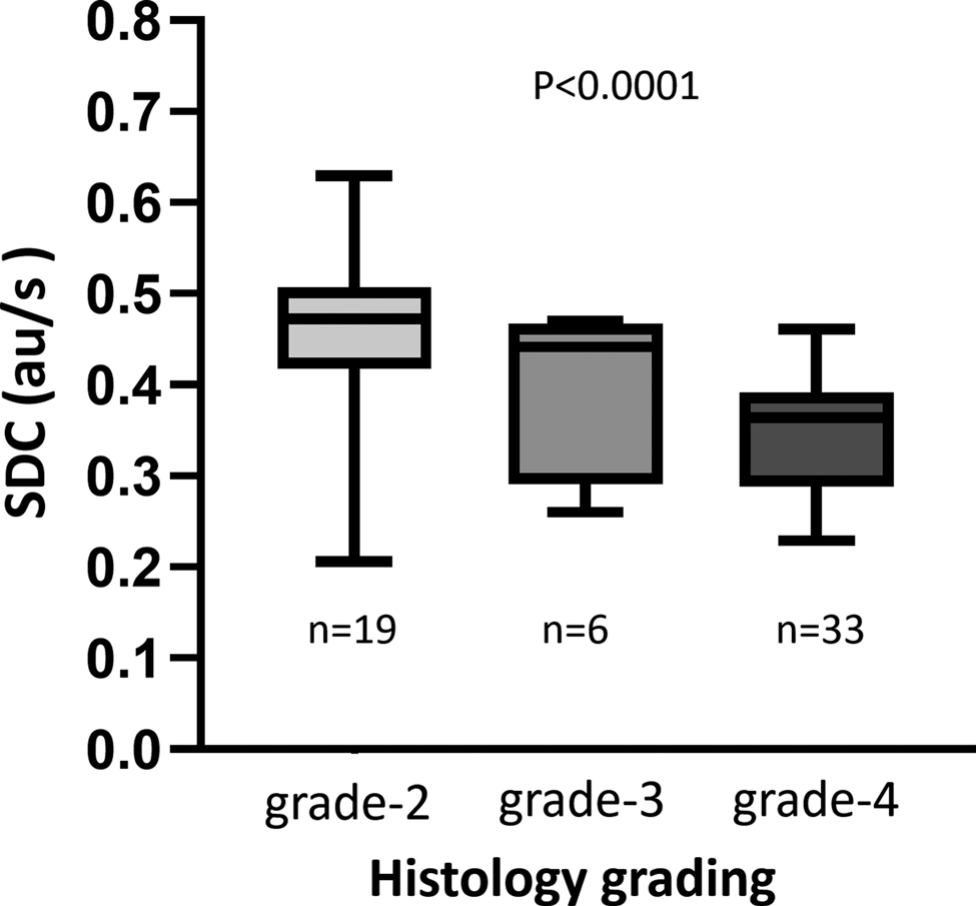
Glioma SDC value Box and Whiskers plots. Tumor histological grade was significantly correlated with SDC, with higher-grade tumors exhibiting lower SDC values. As the tumor grade progressively increased from grade 2 to grade 4, SDC demonstrated a stepwise downward trend

There was a statistically significant difference among the three histological grading of glioma (median value: 0.472 au/s for grade-2 tumors, 0.441 au/s for grade-3 tumors, 0.364 au/s for grade-4 tumors, *p* < 0.0001 among the groups, Fig. 3).

**Fig. 4:**
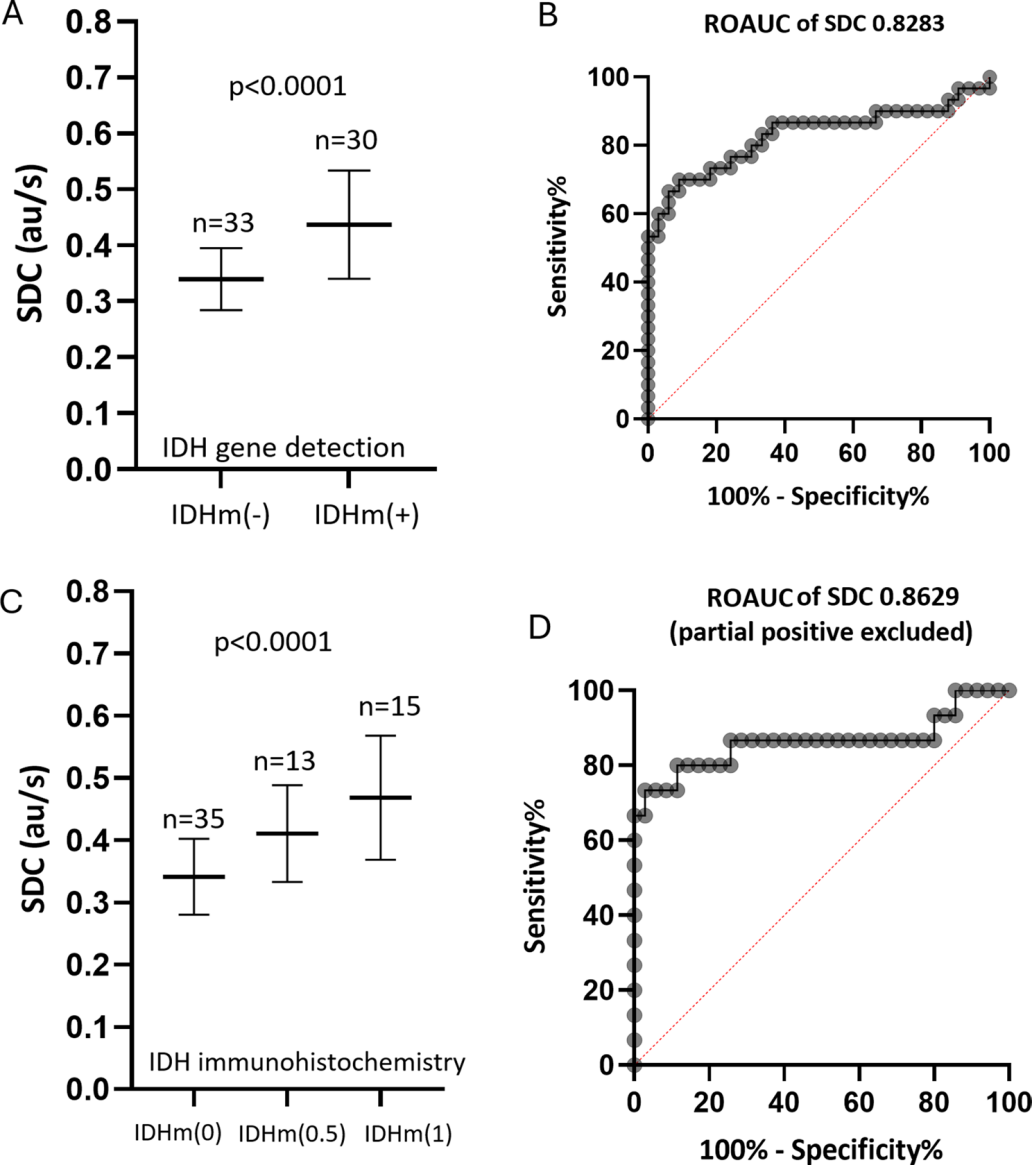
IDH mutant negative tumors had lower SDC values and IDH mutant positive gliomas had higher SDC values. Data **A** and **B** are based on IDH gene testing results. Data **C** and **D** are based on IDH immunohistochemistry results. In D, partially positive tumors were excluded. IDHm (-) or IDHm (0): IDH mutant negative; IDHm (+) or IDHm (1): IDH mutant positive; IDHm (0.5): IDH mutant partially positive

Based on IDH gene testing, IDH mutant negative tumors (*n* = 33) had SDC value of 0.339 ± 0.055 (mean ± standard deviation) au/s, IDH mutant positive tumors (*n* = 30) had SDC value of 0.437 ± 0.097 au/s (Fig. 4A). ROC analysis shows IDH mutant negative and positive tumors were separated with an AUC of 0.828 (Fig. 4B). SDC of > 0.404 au/s had a specificity of 91% and sensitivity of 70% in diagnosing IDH mutant status for glioma (Table 2). Based on IDH immunohistochemistry assessment, IDH mutant negative tumors (*n* = 35) had SDC value of 0.341 ± 0.061 au/s, IDH mutant partially positive tumors (*n* = 13) had SDC value of 0.411 ± 0.078 au/s, and IDH mutant positive tumors (*n* = 15) had SDC value of 0.469 ± 0.10 au/s (Fig. 4C). If partially positive tumors were not included, ROC analysis shows immunohistochemistry IDH negative and positive tumors can be separated with an AUC of 0.863 (Fig. 4D). SDC of > 0.40 au/s had a specificity of 86% and sensitivity of 80% in diagnosing IDH mutant status for glioma (Table 2).

**Fig. 5:**
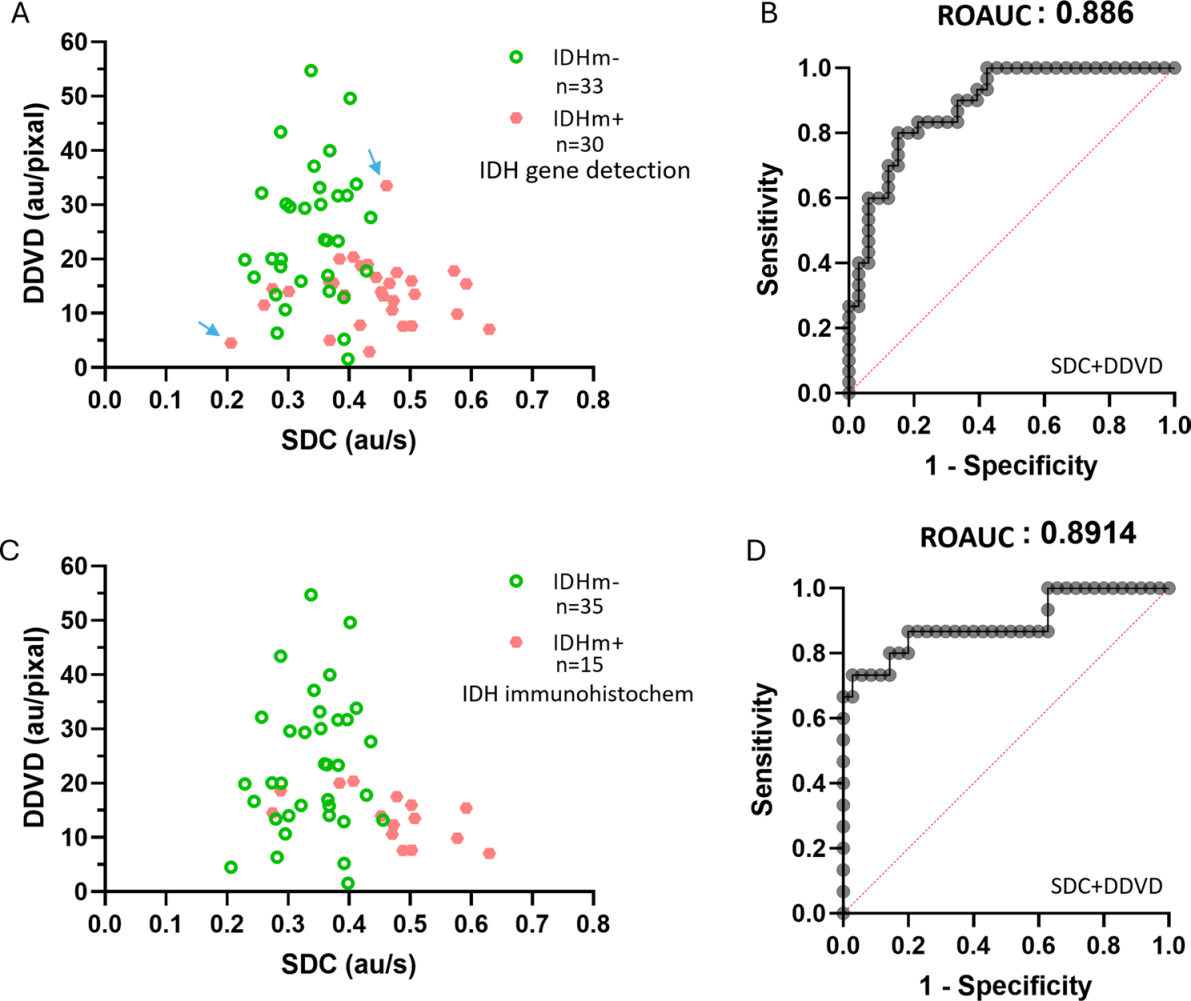
Separating IDH mutant negative (IDHm-) and positive (IDH+) gliomas by a combination of SDC and DDVD. **A** and **B** include all 63 cases, and IDH results were based on gene testing. In **C** and **D** IDH, results were based on immunohistochemistry, and partially positive cases were removed. A comparison of A and C shows, IDH mutant partially positive cases were more likely to have diffusion metrics deviated from the clustering of IDH mutant positive cases and the clustering of IDH mutant negative cases (for example, the two cases labeled with arrow in A). ROC: Receiver operating characteristic curve

SDC was negatively and weakly correlated with DDVD, with Pearson *r* of -0.212 (95% CI: -0.436~-0.038, *p* = 0.096). ROC analysis shows a combination of SDC and DDVD separated IDH mutant negative and positive tumors (based on gene testing) with an AUC of 0.886 (Fig. 5A, B). If partially positive tumors were not included, ROC analysis shows a combination of SDC and DDVD separated immunohistochemistry IDH mutant negative and positive tumors with an AUC of 0.891 (Fig. 5C, D).

**Fig. 6:**
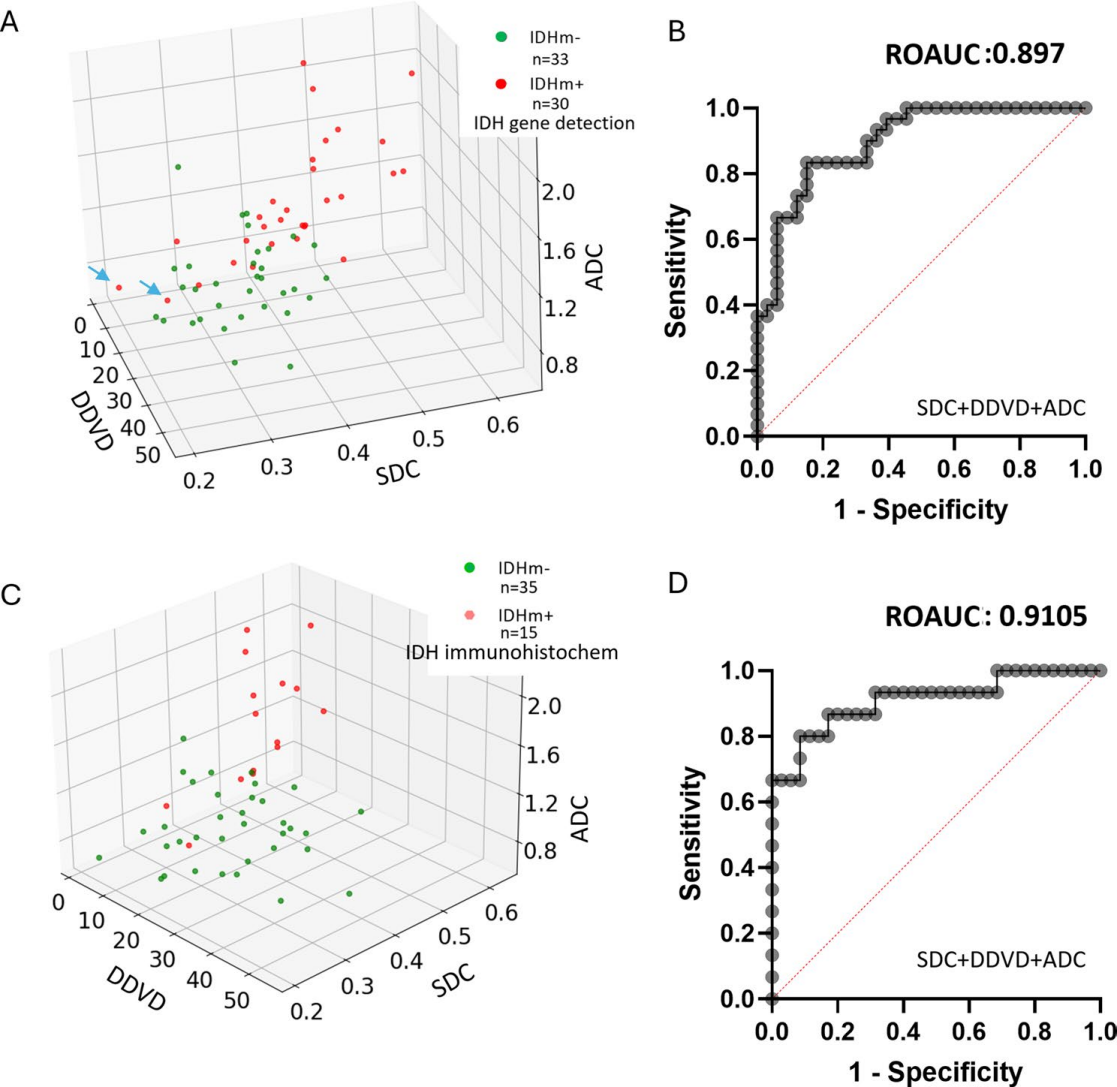
Separating IDH mutant negative (IDHm-) and positive (IDHm+) gliomas by a combination of SDC, DDVD, and ADC. **A** and **B** include all 63 cases, and IDH results were based on gene testing. In **C** and **D**, IDH results were based on immunohistochemistry, and partially positive cases were removed. A comparison of A and C shows, IDH mutant partially positive cases were more likely to have diffusion metrics deviated from the clustering of IDH mutant positive cases and the clustering of IDH mutant negative cases (for example, the two cases labeled with arrow in A). SDC in au/s, DDVD in au/pixel, ADC in 10^− 4^ mm^2^/s

SDC was positively and moderately correlated with ADC, with Pearson *r* of 0.705 (95% CI: -0.554~-0.811, *p* < 0.0001). ROC analysis shows a combination of SDC, DDVD, and ADC separated IDH mutant negative and positive tumors (based on gene testing) with an AUC of 0.897 (Fig. 6A, B). If partially positive tumors were not included, ROC analysis shows a combination of SDC, DDVD and ADC separated immunohistochemistry IDH mutant negative and positive tumors with an AUC of 0.911 (Fig. 6C, D).

According to the data in Fig. 6A, the probability of a tumor being IDH mutant positive is modelled as:

4 $${\text{probability}}\, = \,1/\left[ {1 + exp - \left( \begin{gathered}8.941*SDC - 0.124*DDVD \hfill \\+ 0.034*ADC - 1.229 \hfill \\\end{gathered} \right)} \right]$$

**Fig. 7:**
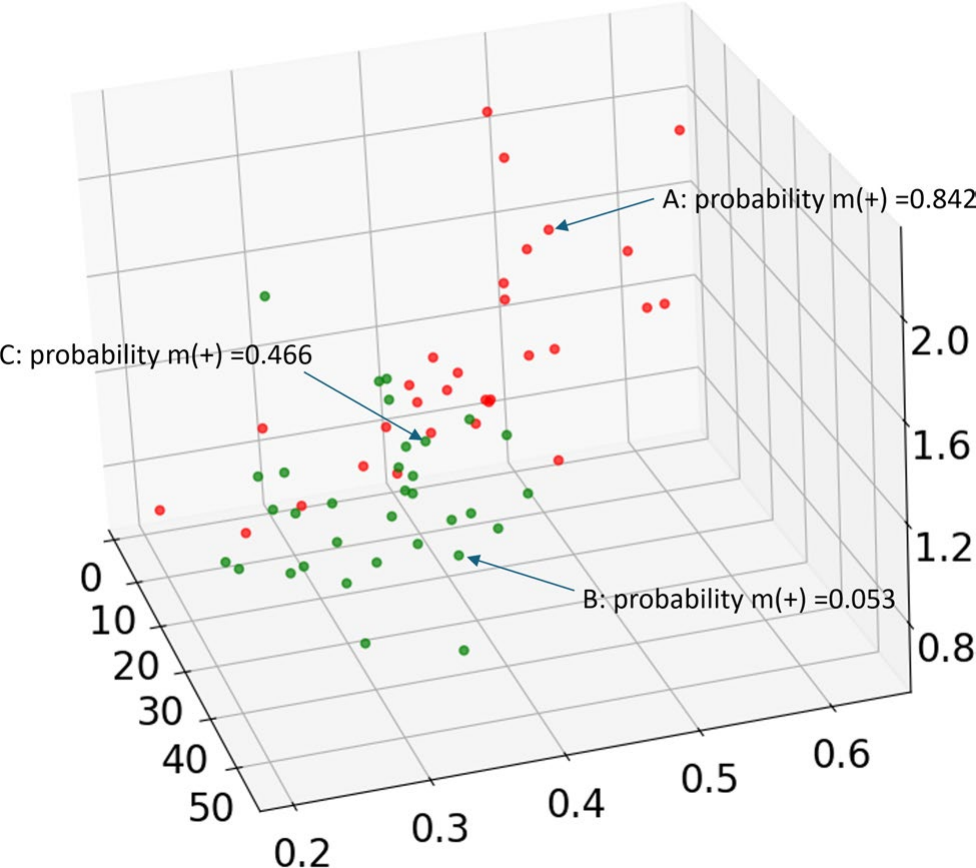
Probability determination of each glioma being IDH mutant positive or negative by a combination of SDC, DDVD, and ADC. This figure is the same as Fig. 6A. According to Eq. (4), the dot of A, B, C has the probability of 0.842, 0.053, and 0.466, respectively, for being IDH mutant glioma [m(+)]

According to Eq. (4), the dots of A, B, C had the probability being IDH mutant positive of 0.842, 0.053, and 0.466, respectively (Fig. 7).

## Discussion

The current study is the first to test the novel MRI diffusion metric SDC in the evaluation of brain gliomas. The results of this study show, glioma grade was significantly correlated with SDC, with higher-grade tumors exhibiting lower SDC values. As the tumor grade progressively increased from grade 2 to grade 4, SDC demonstrated a stepwise downward trend. According to the 2021 WHO classification of central nervous system tumors, glioblastomas constitute the majority of IDH wild-type gliomas. Pathologically, glioblastomas are characterized by two typical characteristics: microvascular proliferation and palisading necrosis [20, 21]. The current study showed that the SDC values were significantly lower in IDH wild-type gliomas compared to IDH mutant gliomas, suggesting slower tissue diffusion in the IDH wild-type gliomas. ROC analysis shows IDH mutant negative and positive tumors (based on gene testing) can be separated with an AUC of 0.828. For IDH immunohistochemistry results, we noted that IDH partially positive glioma had SDC values lying between those of IDH mutant negative gliomas and IDH mutant negative gliomas (Figure-4 C), suggesting an intermediate cellular density and corresponding histopathological profile. Partially positive IDH most likely arise from marked intra-tumoral heterogeneity. Classifying these equivocal cases as the IDH-mutant group increases overlap between the two groups, attenuates between-group differences, and lowers the AUC. Conversely, if partially positive tumors were not included, ROC analysis shows immunohistochemistry IDH negative and positive tumors can be separated with a higher AUC of 0.863. Thus, partially positive cases dilute the distinction between IDH-mutant and IDH-wild-type groups. Moreover, a combination of SDC and DDVD, or a combination of SDC, DDVD, and ADC, further improved the differentiation of IDH mutant negative and positive gliomas. It is interesting to note that, those gene testing IDH positive gliomas showing ‘*atypical*’ diffusion metrics values tend to be IDH immunohistochemistry partial positive cases (Fig.5AC, Fig. 6AC).

SDC was proposed to measure ‘slow diffusion’ [15]. Despite the lower ADC of liver hemangioma than liver cyst, hemangioma shows a very high SDC, and this observation has been shown to be useful for the confirmation of hemangioma MRI diagnosis without contrast agent administration [22]. In the current study, SDC was negatively and weakly correlated with DDVD, suggesting the perfusion component might have been satisfactorily removed from SDC measure. SDC was positively and moderately correlated with ADC, with Pearson *r* of 0.705. Glioma mostly have a T2 relaxation >70 ms [23]. Therefore, it is expected that the information provided by SDC and by ADC may partially overlap [11, 12]. It has been reported that IDH wide-type glioma has longer T1 and shorter T2 [24], thus being explained by denser cells and less ‘watery’. This study shows, despite the higher perfusion and the higher likelihood of necrosis, tissue diffusion was slower in IDH wild-type gliomas. IDH mutant-type glioma has longer T2 and shorter T1 [24], being explained by less dense cells and more ‘watery’. On the other hand, the correlation between SDC and ADC is likely to be tissue-type specific, as noted in the introduction, SDC and ADC can derive very different results for the analysis of liver lesions [15, 19]. Overall, we argue that the three metrics of SDC, DDVD, and ADC are mutually complementary, and which metric may play a more dominant role for disease classification may depend on the tissue and disease types. A combination of these three parameters offers a better classification for lesions.

Ki-67 is a biomarker of cellular proliferation, with high Ki-67 index associated with fast tumor tissues proliferation. We have recently demonstrated that Ki-67 is positively correlated with DDVD in glioma and endometrial carcinoma [10, 25], suggesting that faster proliferating tumor recruited a higher extent of blood perfusion and nutrition. The current study demonstrated that the Ki-67 LI was elevated in IDH-wildtype gliomas or glioblastomas. SDC was negatively and moderately correlated with Ki-67 LI. This is likely due to faster growing tumors are associated with denser cells and thus with less tissue diffusion.

Many earlier literatures reported the performance of parametric MRI in classifying IDH mutation status of gliomas. For differentiation of IDH gene status in gliomas, Guo et al. [26] reported a AUC of 0.870 by amide proton transfer (APT) imaging, of 0.677 by diffusion kurtosis imaging. Cui et al. [27] reported a AUC of 0.881 by a combination of T1 weighted sequence, T2 weighted sequence, DWI image, ADC, and contrast enhanced T1 weighted sequence. Han et al. [28] reported a AUC of 0.756 by proton magnetic resonance spectroscopy, of 0.852 by diffusion tensor imaging, and of 0.890 by a combination of conventional MRI, proton magnetic resonance spectroscopy, conventional diffusion tensor imaging, and diffusion tensor imaging histogram. Zhu et al. [29] reported a AUC of 0.775 by MTR_asym_ (magnetization transfer ratio asymmetry), and of 0.858 by Fit-MTR_normref_ (Lorentzian fitting-magnetization transfer ratio normalized by reference signal). For differentiation of IDH gene status in glioblastomas, Xing et al. [30]. reported a AUC of 0.586 by conventional MRI, of 0.703 by ADC, and of 0.886 for contrast-enhanced cerebral blood volume. Our study showed that, for differentiation of IDH gene status in gliomas, SDC alone had a AUC of 0.828, a combination of SDC and DDVD had a AUC of 0.886, and a combination of SDC, DDVD, and ADC had a AUC of 0.897. Thus, our results compare favorably with literature data [26–30]. Note also that, applying a combination of SDC, DDVD, and ADC as non-invasive to classify glioma has many advantages. SDC was proposed to measure tissue slow diffusion, while DDVD was proposed to measure tissue fast diffusion (i.e., perfusion). Calculation of SDC, DDVD, and ADC does not require special sequences or special hardware, and no contrast agent administration is involved. Practically only four sets of DWI images are acquired (for example, *b* = 0, 10, 500, and 750 s/mm^2^, with *b* = 0 and 750 s/mm^2^ images used to calculate the ADC), thus the data acquisition is reasonably fast. The image post-process is also straightforward. IVIM has also suggested to separately measure perfusion and diffusion information. However, IVIM metrics are heavily affected by tissue T2 relaxation time [12, 31, 32]. IVIM-PF of spleen have been consistently measured being only half of that of the liver [33], and IVIM-PF of HCC have been consistently measured lower than that of the adjacent liver parenchyma [34, 35], and IVIM-PF has been shown to be higher paradoxical in liver with steatosis than in liver without steatosis [36]. All these are unreasonable results. IVIM technique is also associated with long data acquisition time and data fitting instability [37].

There were some limitations in this study. Firstly, it was a single-center study with a relatively small sample size, especially the low numbers of patients with oligodendroglioma and IDH-mutant grade 4 glioma—which precluded more informative analyses such as the comparison of oligodendroglioma vs. astrocytoma and GBM vs. IDH-mutant grade 4 glioma. We will expand the sample size and continue to refine these analyses in future studies. Secondly, DWI scan parameters can be further optimized. An even smaller second *b*-value, such as being smaller than 10 s/mm^2^, is preferred for DDVD analysis. Our earlier study showed that DDVD_b0b2_ is better in assessing HCC than DDVD_b0b10_ [34], and DDVD_b0b5_ is better in assessing rectal carcinoma than DDVD_b0b10_ [38], which is consistent with the initial definition of DDVD [7]. While in this study b-value of 500 and 750 mm²/s were applied, what would be the optimal *b*-values for glioma classification by SDC was also not studied, and may be explored in the future. The NEX of DWI in this study was only 1. Higher NEX will improve image signal and allow better image stability. More advanced DWI sequences, such as ZOOMit (zonal oblique multislice) technique and multiplexed sensitivity-encoding (MUSE) technique etc. can be applied to improve DWI image quality [39, 40]. ZOOMit technique applies two spatially selective parallel excitation pulses to focused sampling, allow for (1) higher resolution imaging of the targeted area, (2) minimized susceptibility-induced artifacts, and (3) shorter echo train lengths. We anticipate that these advanced DWI sequences can further improve glioma classifications. In addition, although the present work focused on the contrast agent enhanced core of the tumors to ensure technical homogeneity, diffusion characteristics of non-enhancing tumor parenchyma and peritumoral edema may also encode IDH-related signatures. Future studies will expand the ROI scheme to include multi-compartment delineations and whole-tumor histogram or radiomic analyses, systematically comparing the discriminative power of each zone and correlating findings with histopathological infiltration margins.

In conclusion, SDC, an in vivo measure of tissue slow diffusion, can help to distinguish between IDH genotypes in diffuse gliomas, aiding in personalized treatment strategies. A combination of SDC and DDVD, or a combination of SDC, DDVD, and ADC, can further improve disease classification. Future studies with more optimized DWI scan parameters and with larger sample sizes for various types of gliomas are required to confirm the results in the current study.

## Data Availability

The datasets used and analysed during the current study are available from the corresponding author upon reasonable request.

## Abbreviations

DDVD: Diffusion-derived vessel density
DWI: Diffusion-weighted imaging
Ki-67 LI: Ki-67 labeling index
FLAIR: Fluid attenuated inversion recovery
FSE: Fast spin-echo
ICC: Intra-class correlation coefficient
IDH: Isocitrate dehydrogenase
NEC: Not elsewhere classified
SDC: Slow diffusion coefficient
T2WI: T2-weighted imaging
ROC: Receiver operating characteristic curve
ROI: Region of interest
WHO: World Health Organization
